# CRISPR/Cas9-mediated precise genome modification by a long ssDNA template in zebrafish

**DOI:** 10.1186/s12864-020-6493-4

**Published:** 2020-01-21

**Authors:** Haipeng Bai, Lijun Liu, Ke An, Xiaochan Lu, Michael Harrison, Yanqiu Zhao, Ruibin Yan, Zhijie Lu, Song Li, Shuo Lin, Fang Liang, Wei Qin

**Affiliations:** 1 Laboratory of Chemical Genomics, School of Chemical Biology and Biotechnology, Peking University Shenzhen Graduate School, Shenzhen, 518055 China; 2 Guangdong Provincial Key Laboratory for Healthy and Safe Aquaculture, Institute of Modern Aquaculture Science and Engineering, School of Life Sciences, South China Normal University, Guangdong, 510631 People’s Republic of China; 3 Department of Molecular, Cell and Developmental Biology, University of California, Los Angeles, CA 90095 USA; 4 Department of Surgery, Keck School of Medicine, University of Southern California, Los Angeles, CA 90033 USA; 5 The Saban Research Institute and Heart Institute of Children’s Hospital Los Angeles, Los Angeles, CA 90027 USA

**Keywords:** CRISPR/Cas9, Homology-directed repair, Long single-stranded DNA, Next-generation sequencing, Disease modeling, Genome editing, Zebrafish

## Abstract

**Background:**

Gene targeting by homology-directed repair (HDR) can precisely edit the genome and is a versatile tool for biomedical research. However, the efficiency of HDR-based modification is still low in many model organisms including zebrafish. Recently, long single-stranded DNA (lssDNA) molecules have been developed as efficient alternative donor templates to mediate HDR for the generation of conditional mouse alleles. Here we report a method, zLOST (zebrafish long single-stranded DNA template), which utilises HDR with a long single-stranded DNA template to produce more efficient and precise mutations in zebrafish.

**Results:**

The efficiency of knock-ins was assessed by phenotypic rescue at the *tyrosinase* (*tyr*) locus and confirmed by sequencing. zLOST was found to be a successful optimised rescue strategy: using zLOST containing a *tyr* repair site, we restored pigmentation in at least one melanocyte in close to 98% of albino *tyr*^*25del/25del*^ embryos, although more than half of the larvae had only a small number of pigmented cells. Sequence analysis showed that there was precise HDR dependent repair of the *tyr* locus in these rescued pigmented embryos. Furthermore, quantification of zLOST knock-in efficiency at the *rps14*, *nop56* and *th* loci by next generation sequencing demonstrated that zLOST showed a clear improvement. We utilised the HDR efficiency of zLOST to precisely model specific human disease mutations in zebrafish with ease. Finally, we determined that this method can achieve a germline transmission rate of up to 31.8%.

**Conclusions:**

In summary, these results show that zLOST is a useful method of zebrafish genome editing, particularly for generating desired mutations by targeted DNA knock-in through HDR.

## Background

Zinc-finger nucleases (ZFNs), transcription activator-like effector nucleases (TALENs) and the clustered regularly interspaced short palindromic repeat (CRISPR) system have been widely used as genome-editing tools in many species, including zebrafish (*Danio rerio*) [1–3]. All three methods engineer DNA through inducing double-strand breaks (DSBs) at specific genomic loci that can be repaired via two major repair pathways: non-homologous end joining (NHEJ) and homology-directed repair (HDR). The NHEJ repair pathway, which is the most common mechanism for DSB repair, directly connects the cut ends leading to insertion/deletion (indel) mutations at high frequency [4].

The CRISPR/Cas9 system has been efficiently used to achieve loss-of-function gene knockout in zebrafish with mutagenesis rates as high as 75–99% [5, 6]. Additionally, knock-in genome modifications, such as single nucleotide polymorphism (SNP) exchange, insertion of small affinity tags (HA, FLAG) or sequences such as loxP elements, can be achieved with the addition of a homologous donor template through HDR [7, 8]. However, such HDR-mediated knock-in approaches for genome editing have proved inefficient in zebrafish [9].

It is possible to knock-in DNA sequences at specific loci through CRISPR/Cas9-mediated NHEJ in zebrafish, however NHEJ-based editing is not precise and the junctions between donors and break points are unpredictable [4, 10]. Precise integration by HDR using a long double-stranded DNA donor (dsDNA) or single-stranded DNA oligonucleotides (ssODN) as homology repair templates has been achieved in zebrafish [2, 7, 9, 11]. Recent work indicated that a plasmid DNA donor produced the highest efficiency among three different donors tested (ssDNA, dsDNA and plasmid) [7, 12]. However, the incorporation of restriction enzyme site efficiency was still only ~ 5% (8 out of 186 fish) [2], and subsequently just a single founder (1/46) was identified for the smaller template and three founders (3/77) for the longer template, both using ssODN to introduce point mutations [13]. Anti-sense asymmetric oligo design was also found to be possible in zebrafish achieving around 2% efficiency of correct HDR knock-in as assessed by high-throughput sequencing analysis [14].

Using a “base editing” (BE) system and zABE7.10 to induce point mutations in zebrafish in our lab, we were able to achieve base substitution at an efficiency between ~ 9–28% with low indel formation. However, these approaches can only introduce base conversion of “C to T” or “A to G”, and the optimal deamination sites for these systems are limited to the CRIPSR/Cas9 target sites [15].

The template donors and target sites for HDR knock-in have varied widely, the latest iteration of which is a recently published method called *Easi*-CRISPR (Efficient additions with ssDNA inserts-CRISPR). *Easi*-CRISPR has been developed in mice as an efficient one-step method for the generation of a targeted DNA insertion with high efficiency [16]. This strategy used long single-stranded DNA (lssDNA) donors with pre-assembled crRNA + tracrRNA + Cas9 ribonucleoprotein (ctRNP) complexes for two CRISPR-Cas9 sites at a single locus in order to generate correctly targeted conditional and insertion alleles in 8.5–100% of the resulting live offspring. As such, this method can overcome the limitations of the other systems discussed above, but whether this strategy can be applied to the zebrafish model and how it compares to other DNA donors is still unknown.

In this manuscript, we report our method, “zebrafish long single-stranded DNA template” (zLOST). This approach is similar to the ssODN methods widely used in the field, although it uses longer single-stranded oligos. We generated a zebrafish *tyrosine* (*tyr*) mutant model (*tyr*^*25del/25del*^*)*, which appears pale because of an inability of melanophores to produce melanin. Subsequently, we used this to visually assess the efficiency of HDR-mediated correction by the re-appearance of pigmented cells in mutant larvae subjected to different genome editing techniques. By scoring the phenotypic rescue of *tyr*^*25del/25del*^ larvae we showed that zLOST could repair the *tyr* mutation considerably more efficiently than previously described approaches. The frequency of rescue/knock-in appeared to be enhanced by one order of magnitude from 5 to 98.5%. To test whether the HDR sequence modification was stably maintained in zebrafish somatic tissue, we used a restriction enzyme-based method and next generation sequencing (NGS) to test efficiency at three other sites (*rps14*, *th* and *nop56*). Quantification of zLOST knock-in efficiency by next generation sequencing demonstrated that we achieved precise genome modification and its application resulted in over a dozen fold increased HDR efficiency in zebrafish. Finally, we were able to recapitulate altered proteins as observed in human diseases with introduction of an exact human mutation at two loci (*twist2* E78Q and *rpl18* L51S). Overall, we demonstrate that zLOST provides a simple and efficient method for inducing precise mutations in zebrafish.

## Results

### Generation of a *tyr* loss-of-function mutant by CRISPR-Cas9

**Fig. 1:**
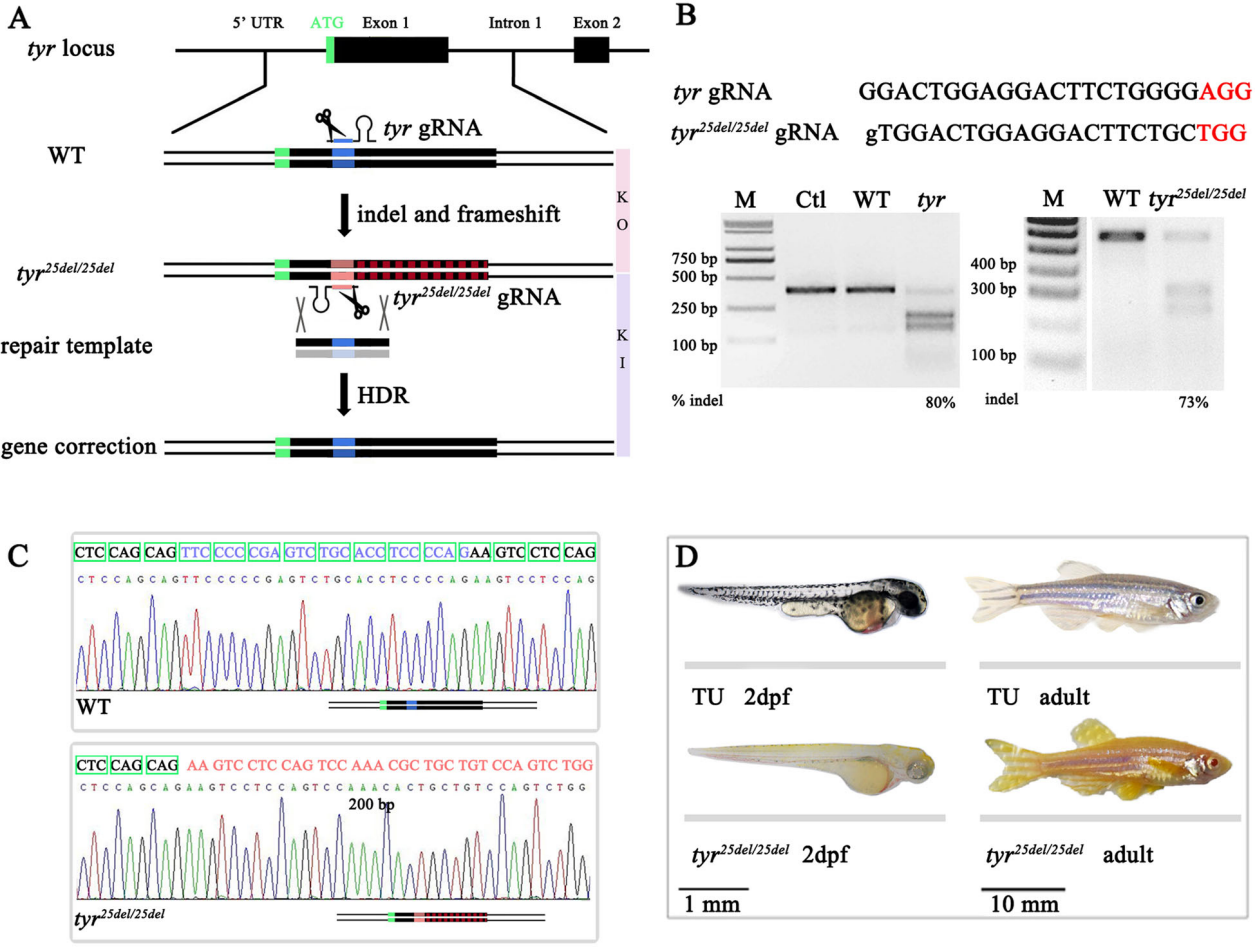
CRISPR-mediated *tyr* knockout to establish a visual knock-in assay. **a** Schematic illustration of CRISPR/Cas9-mediated gene editing of *tyr*. First to knock out (KO) gene function with a 25 nt deletion within the first exon and then knock-in (KI) rescue of this gene using a repair template. **b** Target sites and T7E1 assays of *tyr* and *tyr*^*25del/25del*^ loci. PAMs are marked with red. Ctl represents PCR products without T7E1 digestion. WT denotes PCR products from uninjected embryos with T7E1 digestion. *Tyr* or *tyr*^*25del/25del*^ denotes PCR products from injected embryos with T7E1 digestion. **c** T-cloning and Sanger sequencing identify *tyr*^*25del/25del*^ in F2 zebrafish. Upper row shows wild type (WT) sequence. Open reading frame codons are demarked in green frame. 25 bp deletion in homozygous *tyr* mutants is marked with blue in upper row, which leads to a frameshift mutation (marked with red in lower row). **d** Lateral views of larvae at 2 dpf (scale bar = 1 mm) and adult (scale bar = 10 mm): wild type (upper row) and *tyr*^*25de/l25del*^ (lower row).

The efficiency of genome editing is highly variable between loci making comparable assessments of different published methodologies difficult. To address this, we decided to compare the efficiencies of different HDR-mediated gene editing strategies at a single locus. To do this easily and efficiently, it was necessary to create a suitable animal model. The *tyr* gene encodes tyrosinase which converts tyrosine into melanin, and a mutation in *tyr* results in an albino phenotype in zebrafish embryos; therefore we chose the *tyr* mutant as a quick visible read-out [6]. We designed several single guide RNAs (sgRNA) targeting the *tyr* locus and selected one with high efficiency for the following experiments (Fig. 1a, b). Co-injecting this *tyr* guide RNA (gRNA) and Cas9 mRNA into one-cell stage zebrafish embryos caused reduction of pigmentation in more than 96% of injected embryos (108/112), some of which totally lacked pigmentation. A T7E1 mutagenesis assay demonstrated a ~ 80% efficiency of indel mutation at the locus (Fig. 1b). After screening several founders that transmitted targeted indels to F1 progeny, we established a stable line named *tyr*^*25del/25del*^ that has a frameshift mutation caused by the deletion of 25 bp (Fig. 1c). The homozygous *tyr*^*25del/25del*^ adult fish and their embryos developed normally but lacked body pigmentation (Fig. 1d). To verify the reliability of the *tyr*^*25del/25del*^ mutant line, *tyr* transcripts were measured using quantitative real-time PCR (qRT-PCR). At 3 days post fertilization (dpf), *tyr* transcripts were significantly downregulated compared with sibling control embryos (Additional file 1: Figure S1). Since melanophores of *tyr*^*25del/25del*^ are unable to produce melanin, this feature was used as a quick visible read-out for quantitatively comparing multiple repair template donors for HDR because of the correlation between phenotypic rescue and knock-in efficiency.

### Comparison and optimisation of DNA template donors for HDR mediated knock-in efficiency

**Fig. 2:**
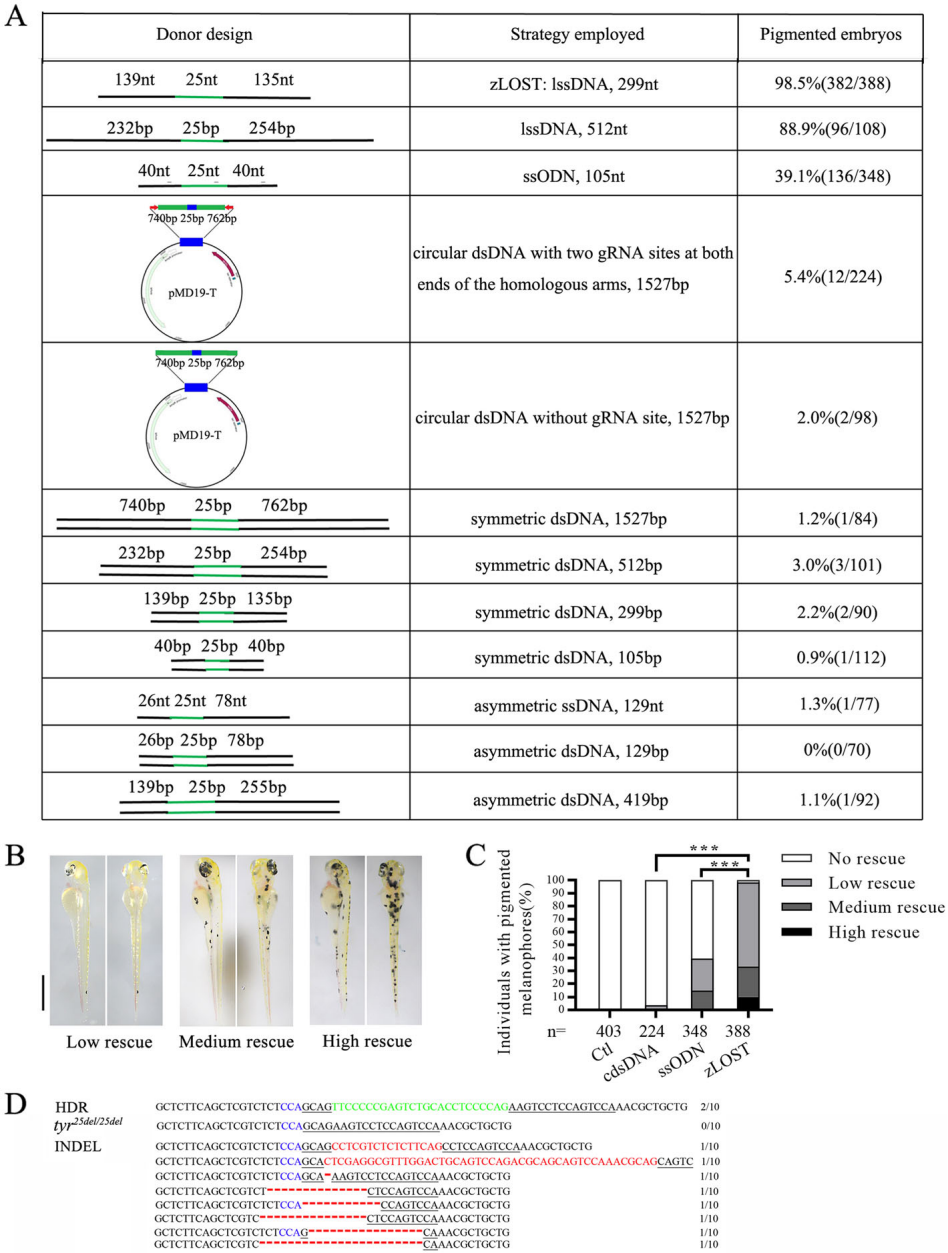
A genetic assay for comparing the efficiency of homology-directed repair using *tyr* mutant. **a** Table of template design schematics (left), attributes of the template (middle) and proportion of observed pigmented embryos in the *tyr*^*25de/l25del*^ model (right). Embryos are analysed at 2 dpf after co-injection of zCas9 mRNA, *tyr*^*25de/l25del*^ gRNA together with repair template. Number of embryos evaluated (n) exceeded 100 for each condition. **b** Phenotypic evaluation of embryos at 2 dpf into three groups according to number of pigmented cells: low rescue (1–20 pigmented cells), medium rescue (21–40 pigmented cells) and high rescue (more than 40 pigmented cells). Scale bar = 1 mm. **c** Statistics of HDR efficiency induced by different repair templates. zLOST: long single stranded template 299 bp, ssODN: single strand DNA oligonucleotides 105 bp, cdsDNA: circular double stranded DNAs 1527 bp (with two gRNA sites at both ends of the homologous arms), Ctl: without repair template. Number of embryos assessed (n) is shown for each group. X^2^-test (***p < 0.001). **d** Sequence analysis confirming that the larvae contained a correctly repaired *tyr* locus by zLOST. Correct insertion by HDR (green), PAM region (blue), target sites (underlined), Indels (red) are indicated.

The 25 bp deletion in the *tyr*^*25del/25del*^ genome created a new CRISPR-Cas9 site, which itself showed 73% efficiency of generating indels, here named *tyr*^*25del/25del*^ gRNA (Fig. 1a, b, Additional file 2: Figure S2). To compare the HDR efficiencies of different strategies, we designed 12 different DNA donors (Fig. 2a). For the circular dsDNA (cdsDNA) donor, the targeted genomic locus of *tyr* was amplified from wild type genomic DNA, and cloned into a pMD19-T vector both with and without two CRISPR target sites at both ends of the homologous arms. We used a symmetrical 105 nt ssODN or an asymmetrical 129 nt ssODN both synthesised by Sangon Biotech. For the zLOST donors, a 299 nt or 512 nt lssDNA containing exon 1 was generated, with the 25 nt flanked by symmetrical left and right homology arms using the following protocol [17]. The dsDNA donor fragments were generated by PCR and purified. Since *tyr*^*25del/25del*^ gRNA could not target the wild type *tyr* sequence, we directly injected zCas9 mRNA, *tyr*^*25del/25del*^ gRNA and the different donors into homozygous albino embryos and checked the rate of pigmentation recovery (Fig. 2a).

If the injected embryos gained pigmentation, we named these individuals “pigmented embryos”. Scoring the pigmented embryos in a *tyr*^*25del/25del*^-HDR assay indicated that variations in length, single- vs. double-stranded DNA, linear vs. circular templates, and symmetrical vs. asymmetrical template donors all affected the HDR efficiency. Our observations suggest that HDR efficiency was maximal across zLOST, ssODN, and cdsDNA donors with two gRNA sites at both ends of the homologous arms (Fig. 2a). However, among dsDNA fragment donors, the length and symmetry of homology arms (≤3%) did not show an order of magnitude difference.

To further validate the high HDR efficiency of zLOST, ssODN, and cdsDNA donors, we compared and quantified the effectiveness of the *tyr*^*25del/25del*^ rescue assay. For the cdsDNA donor, we found that only 5.4% of the larvae showed small numbers of pigmented cells at 2 dpf. For the ssODN donor with homologous arms, 39.1% of the larvae showed some cells with melanin production, a so-called “low rescue” or “medium rescue” phenotype. Asymmetry of homology arms unexpectedly reduced HDR efficiency (1.3%). However, the zLOST donor resulted in up to 98.5% of injected larvae with observable pigmentation at 2 dpf, significantly more than observed using cdsDNA or ssODN (Fig. 2b, c). While a high percentage of animals had a few cells edited, at those low efficiencies it is unlikely that the mutations will be passed on through the germline. Among these embryos, ~ 10% had extensive pigmentation (“high rescue”, more than 40 pigmented cells per larva) that was never observed in embryos rescued by other strategies (Fig. 2c). To establish that there is a direct “phenotype-genotype” relationship, genome extracts from ten embryos with extensive pigmentation after zLOST were confirmed to contain a correctly repaired *tyr* gene by Sanger sequencing (Fig. 2d). However, we did not identify such precise HDR-based repair in embryos recused by the less efficient ssODN donor and cdsDNA donor templates (data not shown). That may be because the low HDR efficiency observed with ssODN and cdsDNA occludes identification of the knock-in event by Sanger sequencing. We also designed a 512 nt lssDNA, but did not observe a significant increase in HDR efficiency despite the increased homologous arm length (Fig. 2a). These results indicated that using the *tyr* mutant as a quick visible read-out model to assess HDR efficiency was efficient, and that zLOST greatly improved HDR efficiency at the *tyr* locus.

### High-efficiency editing of other genomic sites using zLOST

**Fig. 3:**
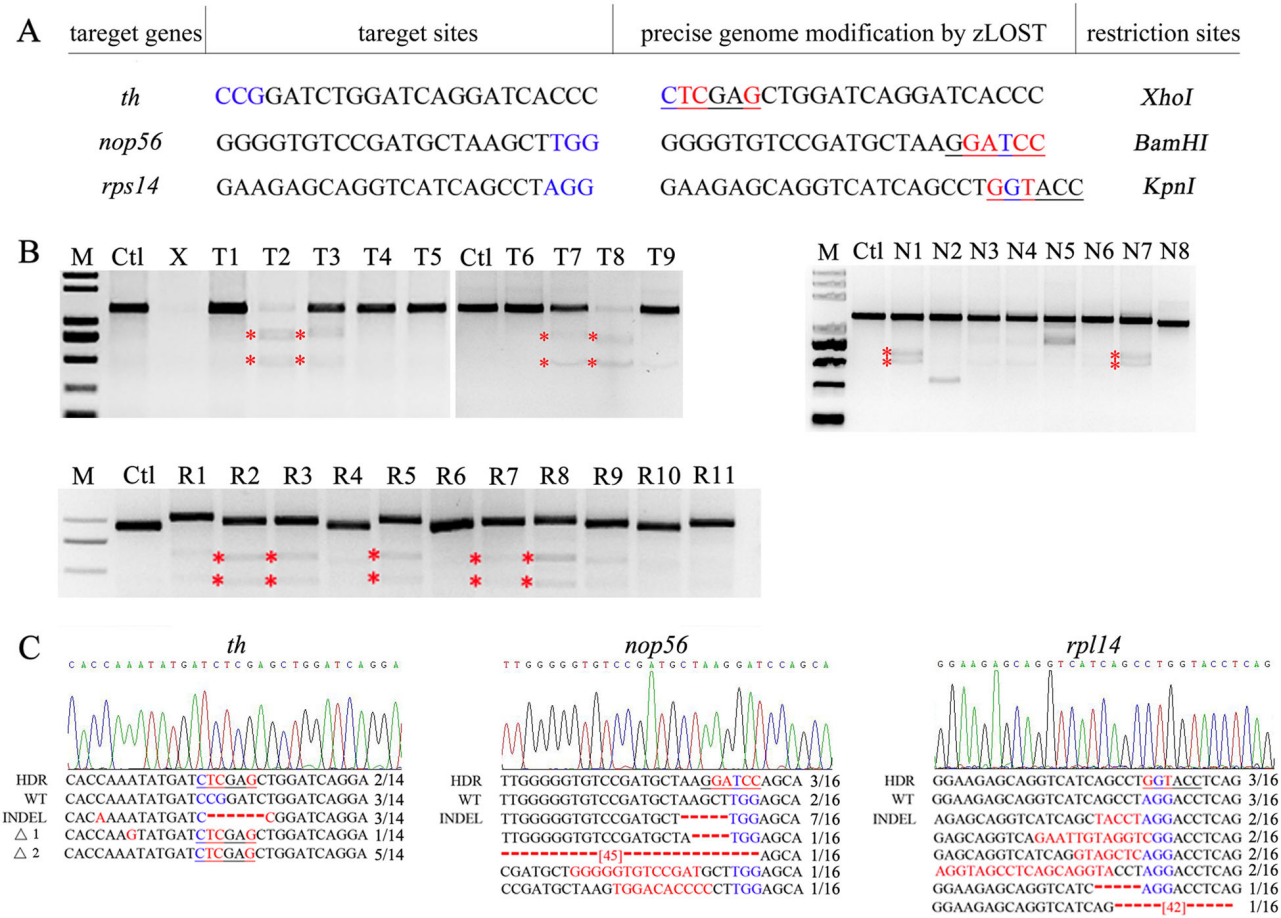
Zebrafish genome editing at three other target sites by zLOST **a** Restriction enzyme-based method design of three target sites. Target sequence (black), PAM region (blue), target modification sites (red), and restriction site (underlined) are indicated. **b** Restriction enzymes are used to digest the amplified region of the target genes. T = *th*, *N* = *nop56*, *R* = *rps14*. The “positive embryos” groups are highlighted by asterisk. **c** Sequencing results of the *th, nop56* and *rps14* loci. Patterns of DNA modification observed in independent embryos pool. Note: △1 and △2 mean the presence of additional undesirable mutations outside of the shown sequence window.

The efficiency of genome editing, regardless of the method used, is highly variable between different loci. Encouraged by the results of the *tyr* mutant gene rescue, we next investigated whether the relatively high efficiency of the zLOST method to precisely edit the zebrafish genome was generally applicable to other loci. We selected new target sites within three genes (*th*, *nop56* and *rps14*) to perform specific knock-ins with different templates and confirm that zLOST efficiency is not a site-specific phenomenon. According to *Easi*-CRISPR and our previous result (Fig. 2a), the distal parts of zLOST were optimally designed to have 150 nt symmetrical homology arms. Details of the target genes, lengths of the ssDNA repair templates, homology arms, and sequencing data are shown in Fig. 3a, c and Additional file 3: Table S1. For each targeted locus, a new restriction site was introduced to identify the positive embryos and to easily screen germline transmission (Fig. 3a). At least 24 embryos per gene were assayed for correct targeting; we randomly selected embryos from the same injection group to perform restriction analysis, and three embryos were pooled per sample to make at least eight technical replicates. For the *th* locus, four of 9 injected embryo groups contained the introduced *Xho*I site (“positive embryos”) using zLOST as the repair template (Fig. 3b, top left). However, we did not find “positive embryos” using other knock-in strategies (data not shown). T-A cloning of the zLOST-modified PCR products followed by Sanger sequencing revealed that two out of 14 clones had seamless HDR modification, while three out of 14 clones carried indels (Fig. 3c). It is worth noting that six out of the 14 clones sequenced showed incorrect-HDR knock-in, as they also showed deletions at the target site (KI + indels) (Fig. 3c, where Δ1 and Δ2 are used to represent the indel, which is out of the shown sequence window). We raised mosaic F0 *th* embryos to adulthood and assayed the rate of germline transmission. Only two of the 21 adult fish that mated produced the desired *Xho*I identifiable allele and the germline transmitted mutations were confirmed by Sanger sequencing.

Using a similar approach for *nop56*, two of 8 samples were identified as “positive embryos” (Fig. 3b, top right) of which *BamH*I site conversion was observed in three of the 16 clones (Fig. 3c). Restriction analysis also indicated that *rps14* sites could be efficiently targeted by zLOST (5 of 11 samples, Fig. 3b, bottom), and precision was again confirmed by sequencing (3 out of 16 clones, Fig. 3c). Taken together, these results demonstrate that knock-in zebrafish with specific point mutations can be generated with high efficiency using the zLOST strategy. Finally, we identified 4 *nop56* founders (*n* = 17) with target knock-in mutations in their germline (23.5% germline transmission rate).

### Improved assessment of zLOST-mediated HDR efficiency using next generation sequencing

We have developed a series of quantitative phenotype assays, restriction enzyme-based methods and Sanger sequencing to assess the specificity and efficiency of HDR by different donor constructs. However, none of these methods can truly assess the validity of HDR in depth because of the occultation of low frequency events. To address this we repeated the microinjections with different donors and selected 20 embryos at 2 dpf to perform next-generation sequencing. Using Illumina sequencing restricted to the targeted region, we quantitatively compared the editing efficiency of the three strategies, ssODN, cdsDNA and zLOST. The desired edit was a single base substitution only at the designed sites. However, considering that random mutation could also occur in the vicinity of the gRNA site, we decided that random synonymous mutations, which do not change the encoded amino acid, would not preclude a sample being considered as a correct editing event. For all sequenced samples, we divided the editing events into four categories: WT (no editing events happened), Correct_HDR (correct editing events), Incorrect_HDR (editing events happened, but with undesired events such as indels), and Other (other situations, mainly insertions, deletions and unmapped sequence). There was variation in the percentage of correct HDRs with synonymous mutations because of some unknown processes (Additional file 4: Table S2). Imperfect changes (Incorrect_HDR) were uncommon.

**Fig. 4:**
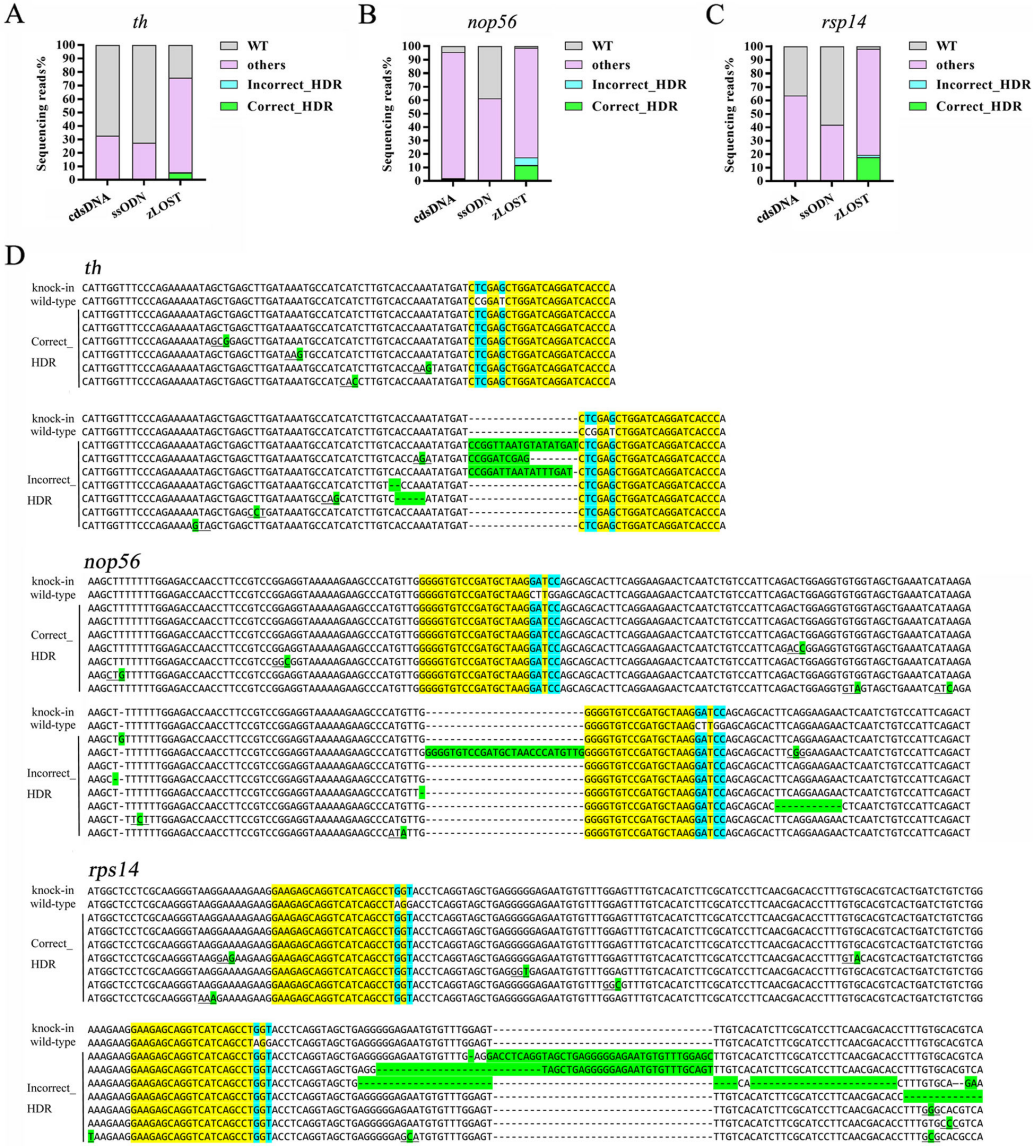
NGS analysis of precise point mutation introduction to the genes *th*, *nop56 and rps14* Total percentages of defined sequence reads classes at knock-in sites of *th* **(a)***, nop56* **(b)** and *rps14* **(c)** genes as engineered with three types of repair template (cdsDNA, ssODN and zLOST). All the reads are divided into four classes: WT, others, correct_HDR and incorrect_HDR. Incorrect_HDR indicates the reads containing target modification sites, but with extra undesirable amino acid changes. **d** Representative examples of different classes of *th*, *nop56* and *rps14* HDR knock-ins: Correct_HDR and Incorrect_HDR.

For gene *nop56*, there were 11,391,197 reads, 10,293,322 reads and 12,240,742 reads obtained from the ssODN, cdsDNA and zLOST samples, respectively. After assembly using FLASH, 97.44, 94.92 and 93.50% of these reads, respectively, were retained. Through analysis of *nop56* editing, the percentage of correct editing events (the Correct_HDR) in zLOST was 11.82%, which was 22-fold higher than in ssODN (0.54%), and 7-fold higher than in cdsDNA (1.62%) (Fig. 4b and d, Additional file 4: Table S2). Similar results were observed for the targeting of the *th* and *rps14* loci (Fig. 4a, c and d, Additional file 4: Table S2). For *th*, Correct_HDR events significantly improved from 0.09% in ssODN-treated embryos to 5.11% in those subjected to zLOST. Similarly for *rps14*, Correct_HDR events were found to be 0.60% in cdsDNA samples, which increased to 17.86% with zLOST. However, unexpected mutations were also found using zLOST, including other point mutations and indels (Incorrect_HDR). Despite this, in the *nop56* loci modified by zLOST, Correct_HDR was still observed twice as frequently as Incorrect_HDR (11.82% vs. 5.64%). As such, the higher percentage of Correct_HDR suggests that our method, zLOST, overall showed a 22 to 57-fold higher editing efficiency than the other strategies.

### zLOST enables precise modelling of human disease mutations in zebrafish

**Fig. 5:**
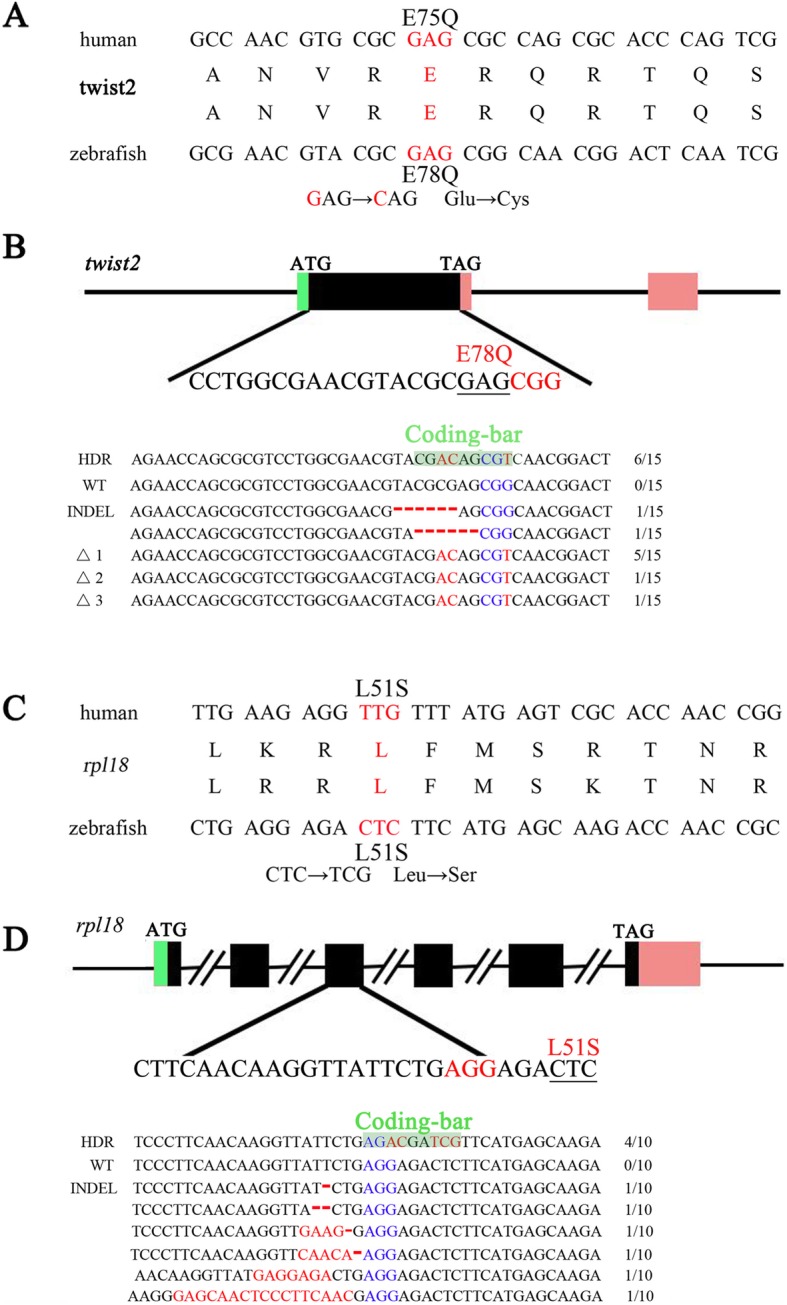
zLOST enables mimicking of human disease related mutations in zebrafish Alignment of human patients and desired zebrafish mutations to model human Barber-Say syndrome (BSS) or Diamond-Blackfan anaemia (DBA), schematic outlines of the gene editing strategy and sequencing of the resulting *twist2* and *rpl18* zebrafish loci. **a** Diagram of the mutation associated with human BSS. The substituted target base is marked in red, which means a p.E78Q amino-acid change in the zebrafish homologue precisely mimics the p.E75Q mutation found in human patients. **b** and **d** Design principles of HDR templates that contain a non-synonymous mutation of the sequence close to the PAM site in addition to synonymous nucleotide changes that create a Coding-bar used for genotyping that utilizes a de novo endonuclease restriction site. Sequencing result at the *twist2* and *rpl18* zebrafish loci targeted by the zLOST system. The Coding-bar includes a restriction endonuclease (*PflFI*) site 5′-GACNNNGTC-3′ in *twist2* and a restriction endonuclease (*PvuI*) site 5′-CGATCG-3′ in *rpl18*. **c** Diagram depicting the mutation associated with human DBA, mimicking the p.L51S mutation at *rpl18* locus found in patients.

Base editing for a single amino acid is crucial to study gene function and model human disease. To this end, we tested the potential of zLOST to introduce human disease-related mutations in zebrafish. In many cases, simple loss-of-function mutations generated by targeted mutagenesis are not sufficient to recapitulate human genetic disorders, particularly diseases arising from gain-of-function point mutations. Clinical studies report specific mutation of *TWIST2* is observed in patients with Ablepharon macrostomia syndrome (AMS) and Barber–Say syndrome (BSS). Both diseases are rare congenital ectodermal dysplasias with similar clinical features, but arise from different mutations: a lysine at TWIST2 residue 75 results in AMS, whereas a glutamine or alanine at the same site yields BSS [18]. We previously used the BE system to induce an amino acid conversion of p.E78K, precisely mimicking the mutation giving rise to AMS in humans [15]. However, the BE system cannot be used to generate a glutamic acid to glutamine change (p.E78Q) as observed in BSS. Instead, we used zLOST to create a p.E78Q mutation in zebrafish (Fig. 5a, b). After co-injecting the *twist2* gRNA, zCas9 mRNA and a lssDNA donor into zebrafish embryos, we found that 4 out of 12 injected embryo sets harboured the desired conversion of G to C (data not shown). Sequencing of the positive embryos successfully detected G to C conversion in 6 out of 15 clones (Fig. 5b). We then went on to identify 7 founders (*n* = 22) with a p.E78Q knock-in mutation in their germline (31.8% germline transmission rate).

We further tested zLOST to generate the mutation of another human disease, Diamond-Blackfan anaemia (DBA), an inherited bone marrow failure syndrome (IBMFS) characterised by erythroid hypoplasia. Recent genetic studies reported that a heterozygous pathogenic non-synonymous variant (p. L51S) of the *rpl18* gene is associated with DBA [19]. To test whether this point mutation directly results in DBA directly, an animal model with the precise point mutation needs to be established. To this end, we successfully used zLOST to achieve the conversion of CTC to TCG in the zebrafish *rpl18* gene, thus inducing a p.L51S amino acid change in this protein (Fig. 5c, d). Further phenotypic analysis will be carried out on the zebrafish as they grow to adults. However, these results provide a clear demonstration of the ability of zLOST to achieve HDR, and the utility of this to transmit precise knock-in alleles through the germline.

## Discussion

HDR-mediated knock-in is a valuable approach for disease modelling and functional analysis. However, the establishment of animal models with specific point mutations is still a challenging task. Although the application of lssDNA donors as a robust method for mouse genome editing has been previously reported [16], a broad application of HDR using lssDNA donors to many loci has yet to be achieved in zebrafish. In this study we present zLOST as an efficient, precise and broadly applicable strategy for generating zebrafish lines with such desired mutations.

Compared with alternatives reported to date [7, 9, 20], zLOST has several advantages: first, unlike a base editing system, we do not have to restrict the editing window to only C to T (or A to G); second, unlike dsDNA, the length of zLOST is only about 300 nt, and so the establishment of zLOST is relatively easy; third, we found that zLOST produced the highest efficiency of the three strategies for HDR on multiple loci. However, one initial concern of zLOST was the relatively high mortality rate and infertility observed in adults, which may be the reason we did not achieve a significant germline transmission rate improvement. zLOST induced high efficiency *tyr* mutant rescue; however, less than 10% of 2 dpf larvae reached adulthood. It was impossible to analyse the rate of germline transmissions when using *tyr*^*25del/25del*^, as the zLOST HDR rescue model showed high lethality and failed to spawn as adults. However, we hypothesised that this may be partly due to complications caused by the *tyr* mutation itself. This appears to have been the case, as we could confirm germline transmission using zLOST targeted to other loci from 9.5 to 31.8% (*th, nop56,* and *twist2*). We also observed some undesired mutations at sites targeted by zLOST; however, these occurred at low efficiency and have also been reported to occur in mice using a similar approach [17]. Such mutations are most likely to arise during ssDNA synthesis, because most reverse transcriptase enzymes do not have proofreading capabilities, so nucleotide misincorporation occurs. To avoid this, the development of reverse transcriptase enzymes with high-fidelity proofreading functions will be helpful.

Recently, several strategies have been reported to improve HDR efficiency in gene targeting [21–23]. The first strategy pertains to the type of donor DNA chosen for targeting. Corn and colleagues reported that asymmetrical ssDNA donors exhibited a 5-fold higher efficiency in vitro than others tested [21]. Antisense asymmetric oligo design was also found to be a successful optimised strategy in zebrafish [14]. We designed asymmetrical ssDNA template and dsDNA templates and tested these in our *tyr*^*25del/25del*^ rescue assay; however, we did not observe improved HDR efficiency using these templates compared with that observed with zLOST (Fig. 2a). Another strategy is to use small molecules to improve HDR efficiency by suppressing the activity of the NHEJ pathway or boosting the activity of the HDR pathway. Recently, the Ge group reported that using Cas9 protein instead of mRNA, suppression of NHEJ with SCR7 and stimulation of HDR pathways with RS-1 in combination could increase the efficiency of germline transmission of point mutations up to 25% in zebrafish [24]. However, these chemical treatments influence the endogenous DNA repair processes and may be toxic during embryonic development as a result [25]. We have not observed improved HDR efficiency of zLOST when used in combination with Cas9 protein, SCR7 or RS-1 (data not shown). This may be because the additional benefit from such approaches is more limited when used in combination with highly efficient strategies such as zLOST, or the benefit is not great enough to overcome the negative effects of their toxicity.

## Conclusions

From this study, we established a quick visible read-out *tyr* mutant zebrafish model, which can be used for preliminary assessment of HDR efficiency with different or multiple strategies of genome editing. By comparing multiple donors, we conclude that zLOST can introduce accurate mutations to mimic human disease in zebrafish more efficiently, and as such can expedite the study of disease mechanisms and development of therapeutics. zLOST solves a major challenge of harnessing CRISPR engineering to edit the zebrafish genome and offers an efficient strategy for creating point mutations.

## Methods

### Zebrafish husbandry

Wild-type Tu line and *tyr* ^*25del/25del*^ zebrafish, maintained in our own laboratory, were used in this study. Wild-type embryos were obtained from group breedings of 10 female and 10 male fish. For *tyr* ^*25del/25del*^ embryos, 6 pairs of fish were used for mating. All zebrafish experiments were approved by Institutional Animal Care and Use Committee of Peking University. Embryos were raised and maintained at 28.5 °C using standard techniques [26]. Embryos less than 3 dpf are treated with sodium hypochlorite to prevent further development. After study, the zebrafish were euthanized following the NIH guidelines for zebrafish euthanasia. The fish were left in the tricaine methane sulfonate solution (MS222, 168 mg/L, which was buffered with sodium bicarbonate to a neutral pH before immersing fish) for at least 20 min following cessation of opercular movement and frozen quickly in liquid nitrogen.

### Preparation of zCas9 mRNA and sgRNAs

zCas9 mRNA was in vitro transcribed from an *XbaI* linearized zCas9 vector using the T3 mMESSAGE mMACHINE kit (Ambion) [5]. All gRNAs templates in this study were prepared using the cloning-independent gRNA generation method [12], and all target sites are listed in Additional file 5: Table S3. All gRNAs were transcribed in vitro using the T7 RiboMax Express Large Scale RNA Production System (Promega), and purified using an RNeasy FFPE kit (Qiagen). Additional file 3: Table S1 lists all the oligos and primers used in this study.

### Preparation of HDR templates

We mainly set out to compare three donor DNA templates: long dsDNA (plasmid, short ssODN and long ssDNA. The long dsDNA donor templates for HDR were generated by PCR on wild type genomic DNA that was then cloned into pMD19-T and mutated precisely using Fast Mutagenesis Kit V2 (Vazyme) to generate a modified locus with two CRISPR target sites at both ends of the homologous arms, similar to that described previously [7]. This construct was also used to produce an RNA transcript using the T7 RiboMAX Express Large Scale RNA Production System and the RNA transcript was purified using mMESSAGE mMACHINE kit (Ambion). The long ssDNA donors were synthesized by reverse transcription from the RNA templates, this RNA template was then digested using RNase H and the remaining ssDNA was run on agarose gel and extracted from the gel slice using a NucleoSpin® Gel and PCR Clean-up kit (Macherey-Nagel). The short ssODN donors with homologous arms were synthesized by Sangon Biotech. The PAM sites or seed sequences in the HDR donors were altered to prevent re-cutting of the desired donor DNA.

### Zebrafish microinjection, T7E1 assays, and sanger sequencing

A solution (1~2 nL) containing 300 ng/μL zCas9 mRNA, 30 ng/μL gRNA and 10–50 ng/μL template DNA was co-injected into early one-cell-stage zebrafish zygotes as previously described [6]. Injected embryos were incubated at 28.5 °C for examination of phenotypes. After 2 dpf, embryos which developed normally were collected for PCR or imaging. Targeted genomic loci were amplified over the length of ssDNA template from genomic DNA, and then the PCR product was cloned into pEASY-T1 vector (Transgene) for Sanger sequencing. The digested samples are analysed through a 2% agarose gel. All experiments were repeated three times.

### Quantitative PCR

Total RNA was extracted in Trizol Reagent (Life Technologies). cDNA was synthesized using PrimeScript™ RT reagent Kit with gDNA Eraser (Takara) following the manufacturer’s protocol. Quantitative real-time PCR was performed using FastStart SYBR Green Master (Roche). Primer sequences are listed in Additional file 3: Table S1. Values of three independent samples (*n* = 20 each sample) are shown.

### Imaging

Zebrafish embryos were anesthetized with 0.03% Tricaine (Sigma-Aldrich), and mounted in 4% methylcellulose. Photographs were taken by a Zeiss Axio Imager Z1 microscope, and processed by Adobe Photoshop CS software.

### Genomic DNA extraction

Samples for genotyping of embryos (2 dpf) were prepared using the HotSHOT method [27]. Briefly, the genomic DNA was extracted from whole embryos incubated in 20 μL NaOH (50 mM) at 95 °C for 20 min. The solution was then cooled to room temperature and neutralized with 2 μL Tris-HCL (1 M, pH 8.0). The genomic DNA samples were mixed and centrifuged, then the supernatants used for genotyping. With multiple replicates carried out, we randomly collected at least 8 sets with 3 embryos per tube for each condition.

### Illumina-based sequencing to quantify knock-in rates (next generation sequencing)

To avoid interference by the residual donor DNA, we designed a pair of primers that are located outside the region of the donor DNA for the first round of PCR, which was purified to continue as the second round template for amplification. Targeted allele analysis was performed by amplifying genomic regions of interest with Q5 High-Fidelity 2× Mastermix (NEB, M0492) using a two-round PCR strategy to add Illumina P5 adaptors (AGATCGGAAGAGCGTCGTGTAGGGAAAGAGTGT) and P7 adaptors (AGATCGGAAGAGCACACGTCTGAACTCCAGTCAC). It is also possible to use the linker sequence contained in the trim data of the quality control software. Libraries were sequenced with 1 × 200-cycle MiSeq runs (Illumina) (Genewiz).

FASTQ files containing paired sequencing reads were assembled by FLASH [28] (https://ccb.jhu.edu/software/FLASH/). After assembling, each read represents a sample because the gene sample for sequencing is only 250 bp length, which is shorter than the length of the unpaired single reads of Illumina. All samples were mapped to the reference gene through sequence alignment by using EMBOSS Needle tool (http://www.bioinformatics.nl/cgi-bin/emboss/needle). Then a Python script was designed categorize the editing events for every sample. The counts of different event categories were processed and plotted by using an R script.

The correct editing event that meets our expectations is, within the range of gRNA, only our desired base substitutions occurred without other substitutions or indel events and the protein translated from the cds of the gene only has the expected residue substitution after editing. Of the region covered by gRNA of the gene, we refer to the reference sequence as WT_sgRNA_pattern, the sequence after correct editing as HDR_sgRNA_pattern. The categorizing of editing events was performed according to the following strategies:

1. If the WT_sgRNA_pattern could match exactly to the sample sequence, we assume that no editing events occurred in this sample and categorized it as “WT”.
2. When the HDR_sgRNA_pattern matched to the sample sequence, we checked to see if the protein sequence coded by the CDS region of the gene only contained expected residue substitutions. If true we regard the editing event occurred in this sample as “Correct_HDR”, if false as “Incorrect_HDR”.
3. If neither WT_sgRNA_pattern or HDR_sgRNA_pattern could match to the sample sequence, we assume that some other event happened to the sample and categorize it as “Others”, which mainly contains insertions, deletions and unmapped sequence.

## Supplementary information

**Additional file 1: Figure S1.** qPCR analysis of the *tyr* expression in *tyr*^*25del/25del*^ zebrafish. Embryos were harvested at 2 dpf. Results are expressed as mean ± S.D. (*n* = 3), ***P<0.001.

**Additional file 2: Figure S2.** Sequencing result of *tyr*^*25del/25del*^ gRNA induced indels.

**Additional file 3: Table S1.** Primers used in this study.

**Additional file 4: Table S2.** High-throughput sequencing analysis of point mutation knock-ins frequency by different donors.

**Additional file 5: Table S3.** Target sites in this study. Red colors means PAM sequences.

## Data Availability

All datasets supporting the conclusions of the manuscript are included within the article and its additional files. Raw data used to generated NGS analysis in this manuscript are available at https://drive.google.com/drive/folders/1_gcDAnFH9Zx01Ka0zmPFc6OJeH9zc33y?usp=sharing.

## Abbreviations

BE: Base editing
cdsDNA: Circular dsDNA
CRISPR: Clustered regularly interspaced short palindromic repeat
DSBs: Double strand breaks
dsDNA: Double strand DNA donor
*Easi*-CRISPR: Efficient additions with ssDNA inserts-CRISPR
HDR: Homology-directed repair
KI: Knock in
lssDNA: Long single-stranded DNA
NGS: Next generation sequencing
NHEJ: Non-homologous end joining
SNP: Single nucleotide polymorphism
ssODN: Single strand DNA oligonucleotides
TALENs: Transcription activator-like effector nucleases
ZFNs: Zinc-finger nucleases
zLOST: Zebrafish long single-stranded DNA template
