# Supplementary figures and images for: CRISPR/Cas9-mediated precise genome modification by a long ssDNA template in zebrafish
Source: BMC Genomics. 2020 Jan 21;21:67. doi: 10.1186/s12864-020-6493-4 (PMC6974980; doi:10.1186/s12864-020-6493-4)

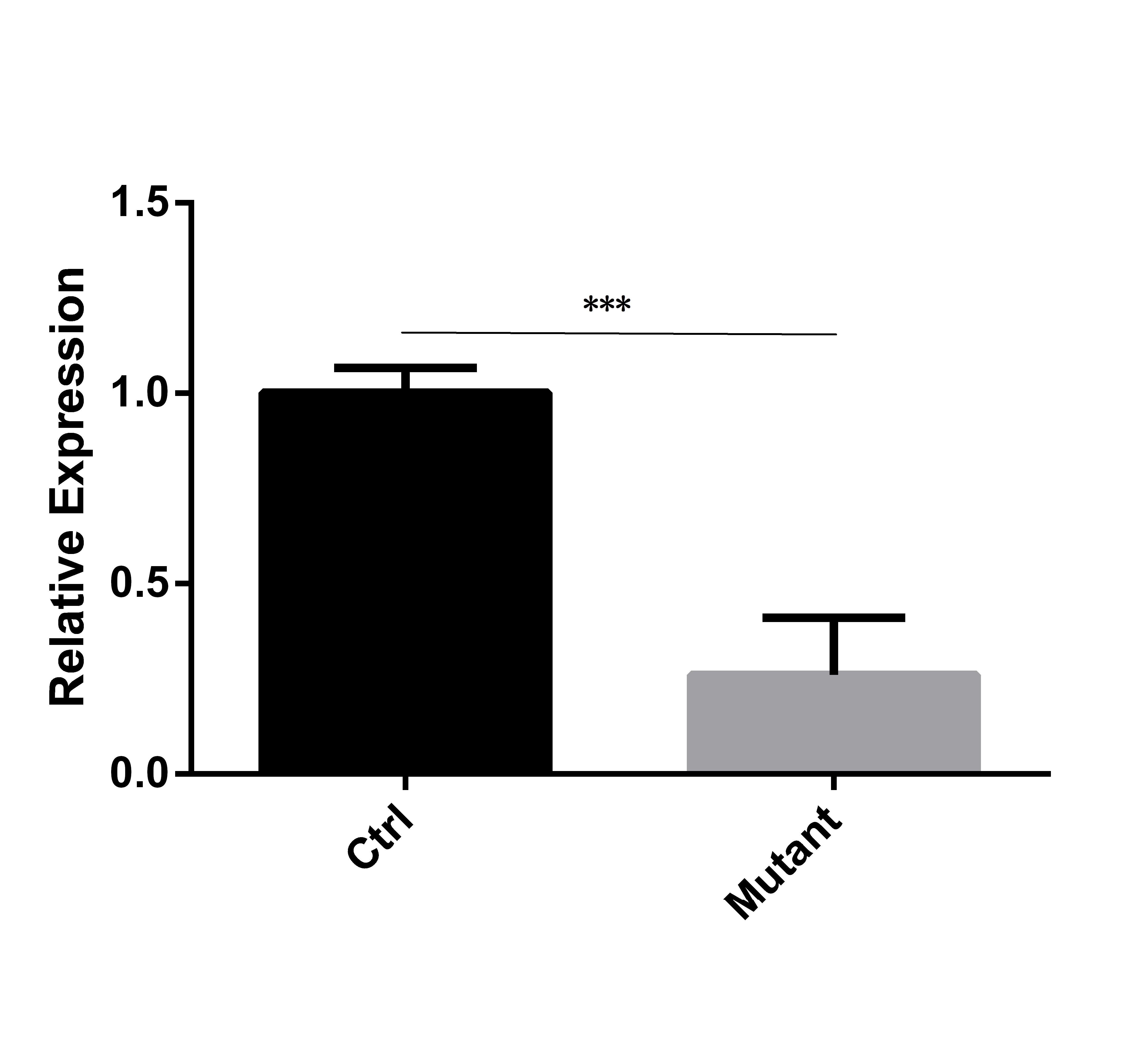

Supplement: Supplementary file 1 — Additional file 1: Figure S1. qPCR analysis of the tyr expression in tyr25del/25del zebrafish. Embryos were harvested at 2 dpf. Results are expressed as mean ± S.D. (n = 3), ***P<0.001. [file 12864_2020_6493_MOESM1_ESM.jpg]

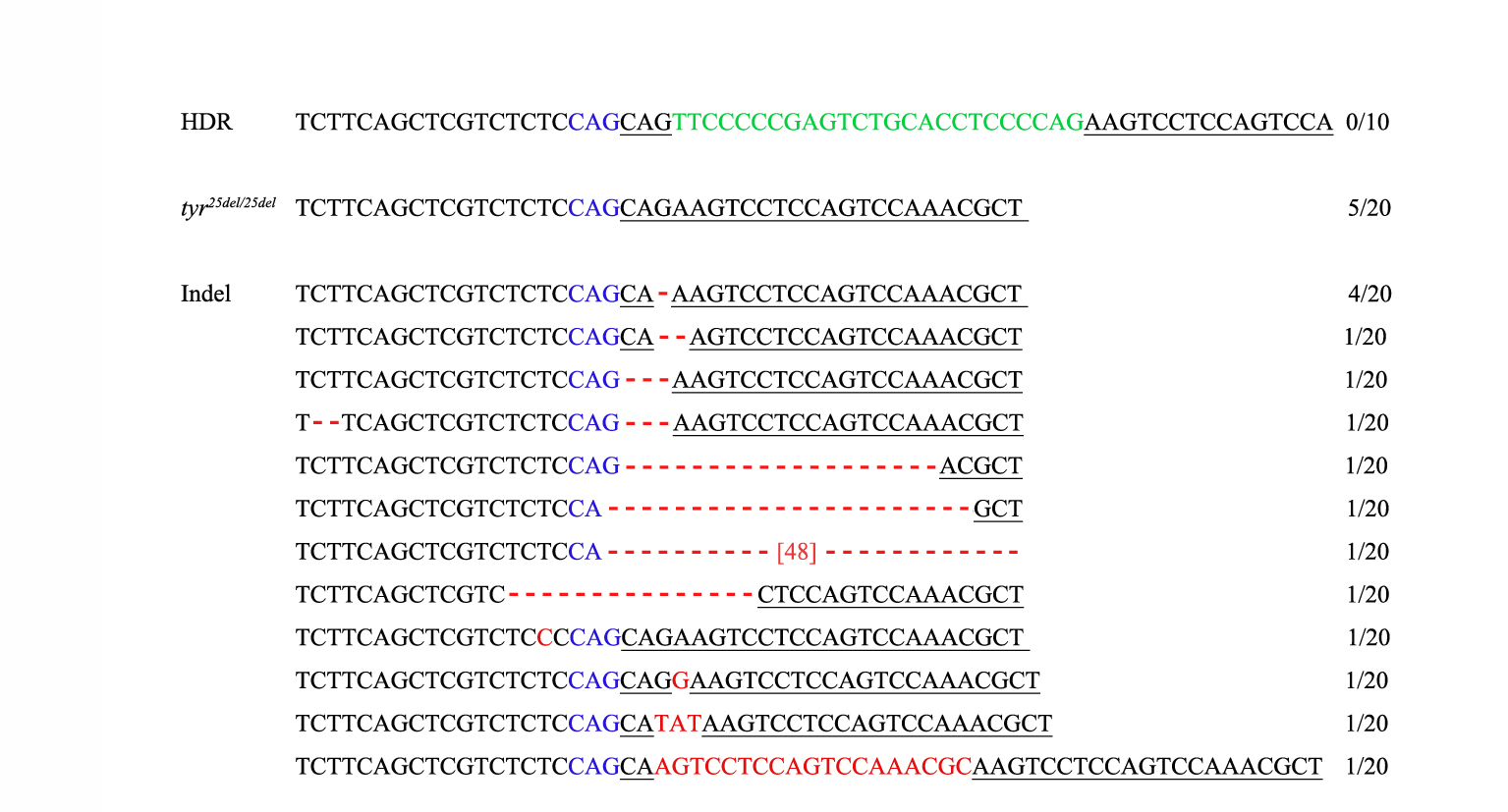

Supplement: Supplementary file 2 — Additional file 2: Figure S2. Sequencing result of tyr25del/25del gRNA induced indels. [file 12864_2020_6493_MOESM2_ESM.jpg]
