# Supplementary material for: CRISPR/Cas9-mediated precise genome modification by a long ssDNA template in zebrafish
Source: BMC Genomics. 2020 Jan 21;21:67. doi: 10.1186/s12864-020-6493-4 (PMC6974980; doi:10.1186/s12864-020-6493-4)
Supplement: Supplementary file 3 — Additional file 3: Table S1. Primers used in this study. [file 12864_2020_6493_MOESM3_ESM.docx]

Table S1. Primers used in this study.

| Name | Sequence of the primer (5’-3’) |
| --- | --- |
| *tyr ^25del/25del^* | TAATACGACTCACTATAGTGGACTGGAGGACTTCTGCGTTTTAGAGCTAGAAATAGC |
| *tyr*-gRNA | TAATACGACTCACTATAGGACTGGAGGACTTCTGGGGGTTTTAGAGCTAGAAATAGC |
| homologous arms1500-F | TTATAAAAGTGTGTGTGTGTGTT |
| homologous arms1500-R | GTCAAATAAAAGTCTGCTTTAC |
| ssODN donor *tyr* | CCTCTTCTTCCTCCAGCTCTTCAGCTCGTCTCTCCAGCAGTTCCCCCGAGTCTGCACGTCCCCAGAAGTCCTCCAGTCCAAACGCTGCTGTCCAGTCTGGCCCGG |
| testing-*tyr*-F | GAGAGAGAGCATGTGAATGGG |
| testing-*tyr*-R | CGCAGTTGGCACCGAAGAAGCCG |
| zLOST-300-*tyr*-F | TAATACGACTCACTATAGGTGTGTGTGAAGCATCTCG |
| zLOST-300-*tyr*-R | CTGAGTGAGGATACTGCGGCC |
| zLOST-500-*tyr*-F | TAATACGACTCACTATAGGTGTGTGTGAAGCATCTCG |
| zLOST-500-*tyr*-R | CGCAGTTGGCACCGAAGAAG |
|  |  |
| *rps14*-gRNA | TAATACGACTCACTATAGAAGAGCAGGTCATCAGCCTGTTTTAGAGCTAGAAATAGC |
| testing-*rps14*-F | CGTGATGTATGTCGTTATCTAGAGATG |
| testing-*rps14*-R | GCATGTGCACACAAAGACAC |
| zLOST-*rps14*-F | TAATACGACTCACTATAGCATCTTGTATTTAATTCTACCATGTCTAATTG |
| zLOST-*rps14*-R | TCAGGATACATACTTGCCAGACA |
| Template-mutation-*rps14*-F | TCATCAGCCTGGTACCTCAGGTAGCTGAGGGGGAGAATGTGTT |
| Template-mutation-*rps14*-R | TACCTGAGGTACCAGGCTGATGACCTGCTCTTCCTTCTTTTCC |
|  |  |
| *th*-gRNA | TAATACGACTCACTATAGGGTGATCCTGATCCAGATCGTTTTAGAGCTAGAAATAGC |
| testing-*th*-F | TGAAATAAGCGTATGGAGAC |
| testing-*th*-R | GTCCAATAACGTCGTCAAAT |
| zLOST-*th*-F | TAATACGACTCACTATAGGTGATTGTGACACTTATTAC |
| zLOST-*th*-R | TAACTGTGTTTGTTATGCAAAAC |
| Template-mutation-*th*-F | AAATATGATCTCGAGCTGGATCAGGATCACCCAGTAAGTGCTGGAT |
| Template-mutation-*th*-R | CCTGATCCAGCTCGAGATCATATTTGGTGACAAGATGATGGCATTT |
|  |  |
| *nop56*gRNA | TAATACGACTCACTATAGGGGTGTCCGATGCTAAGCTGTTTTAGAGCTAGAAATAGC |
| testing-*nop56*-F | GAGTAAATGGCCATTTTC |
| testing-*nop56*-R | TATAATCACAGCTCTGCTTGAG |
| zLOST-*nop56*-F | TAATACGACTCACTATAGTTGATTTGCTATTTCTGACATG |
| zLOST-*nop56*-R | ATTTTTTGTGGGACCTTAAAGTA |
| Template-mutation-*nop56*-F | CGATGCTAAGGATCCAGCAGCACTTCAGGAAGAACTCAATCTGTCC |
| Template-mutation-*nop56*-R | AAGTGCTGCTGGATCCTTAGCATCGGACACCCCCAACATGGGCTTC |
|  |  |
| *twist2*-gRNA | TAATACGACTCACTATAGCCTGGCGAACGTACGCGAGGTTTTAGAGCTAGAAATAGC |
| testing-*twist2*-F | AAGTGGAGATCGTATTTTCTC |
| testing-*twist2*-R | GTTCGGCATTATCAGGACGCG |
| zLOST-*twist2*-F | TAATACGACTCACTATAGTTTCTTTAAAAAAGAAATG |
| zLOST-*twist2*-R | CTAGTGGGACGCAGACATCGAC |
| Template-mutation-*twist2*-F | GTACGACAGCGTCAACGGACTCAATCGCTGAACGAAGCCT |
| Template-mutation-*twist2*-R | TTGACGCTGTCGTACGTTCGCCAGGACGCGCTGGTTCTGG |
|  |  |
| *rpl18*-gRNA | TAATACGACTCACTATAGCTTCAACAAGGTTATTCTGGTTTTAGAGCTAGAAATAGC |
| testing-*rpl18*-F | GGAGTTGACATCAGACACAACAAG |
| testing-*rpl18*-R | CCTGTCTCCCTTGCTAAATATAG |
| zLOST-*rpl18*-F | TAATACGACTCACTATAGGCTGTAAAATAGGTACAAAAC |
| zLOST-*rpl18*-R | CATAACAGCGTGTGTGGTCCGTGAT |
| Template-mutation-*rpl18*-F | ATTCTGAGACGATCGTTCATGAGCAAGACCAACCGCCCAC |
| Template-mutation-*rpl18*-R | CGATCGTCTCAGAATAACCTTGTTGAAGGGAGCATCAGAAC |
|  |  |
| β-actin-F | ATGCCCCTCGTGCTGTTTTC |
| β-actin-R | GCCTCATCTCCCACATAGGA |
| RT-tyr-F | ACTGTCAGGTGTGCACGGAT |
| RT-tyr-R | GCCTCGCGGAGGTTGTAACT |
