# Supplementary material for: CRISPR/Cas9-mediated precise genome modification by a long ssDNA template in zebrafish
Source: BMC Genomics. 2020 Jan 21;21:67. doi: 10.1186/s12864-020-6493-4 (PMC6974980; doi:10.1186/s12864-020-6493-4)
Supplement: Supplementary file 4 — Additional file 4: Table S2. High-throughput sequencing analysis of point mutation knock-ins frequency by different donors. [file 12864_2020_6493_MOESM4_ESM.docx]

Table S2. High-throughput sequencing analysis of point mutation knock-ins frequency by different donors.

| **Gene** | **Group** | **Class** | **Value** | **Frequency** | **%** |
| --- | --- | --- | --- | --- | --- |
| *th* | ssODN | Correct_HDR | 9538 | 0.0008986 | 0.09% |
| *th* | ssODN | Incorrect_HDR | 21307 | 0.002 | 0.20% |
| *th* | ssODN | WT | 7694580 | 0.72493 | 72.49% |
| *th* | ssODN | Others | 2888825 | 0.272165 | 27.22% |
| *th* | cdsDNA | Correct_HDR | 970 | 0.0000855 | 0.01% |
| *th* | cdsDNA | Incorrect_HDR | 166 | 0.0000146 | 0.00% |
| *th* | cdsDNA | WT | 7624246 | 0.67204 | 67.20% |
| *th* | cdsDNA | Others | 3719545 | 0.32786 | 32.79% |
| *th* | zLOST | Correct_HDR | 560082 | 0.051159 | 5.11% |
| *th* | zLOST | Incorrect_HDR | 76658 | 0.007 | 0.70% |
| *th* | zLOST | WT | 2639172 | 0.24107 | 24.11% |
| *th* | zLOST | Others | 7671899 | 0.70077 | 70.08% |
| *nop56* | ssODN | Correct_HDR | 60157 | 0.005419356 | 0.54% |
| *nop56* | ssODN | Incorrect_HDR | 23546 | 0.002121185 | 0.21% |
| *nop56* | ssODN | WT | 4274660 | 0.385090731 | 38.51% |
| *nop56* | ssODN | Others | 6742034 | 0.607368727 | 60.74% |
| *nop56* | cdsDNA | Correct_HDR | 157995 | 0.016170693 | 1.62% |
| *nop56* | cdsDNA | Incorrect_HDR | 50916 | 0.005211222 | 0.52% |
| *nop56* | cdsDNA | WT | 421668 | 0.043157467 | 4.31% |
| *nop56* | cdsDNA | Others | 9139874 | 0.935460618 | 93.55% |
| *nop56* | zLOST | Correct_HDR | 1353283 | 0.11824695 | 11.82% |
| *nop56* | zLOST | Incorrect_HDR | 645282 | 0.05638335 | 5.64% |
| *nop56* | zLOST | WT | 130678 | 0.01141836 | 1.14% |
| *nop56* | zLOST | Others | 9315306 | 0.81395134 | 81.40% |
| *rps14* | ssODN | Correct_HDR | 50780 | 0.00351854 | 0.35% |
| *rps14* | ssODN | Incorrect_HDR | 18263 | 0.00126544 | 0.13% |
| *rps14* | ssODN | WT | 8384486 | 0.58096035 | 58.10% |
| *rps14* | ssODN | Others | 5978585 | 0.41425666 | 41.42% |
| *rps14* | cdsDNA | Correct_HDR | 70868 | 0.006016275 | 0.60% |
| *rps14* | cdsDNA | Incorrect_HDR | 13127 | 0.0011144 | 0.11% |
| *rps14* | cdsDNA | WT | 4277269 | 0.36311489 | 36.31% |
| *rps14* | cdsDNA | Others | 7418064 | 0.62974846 | 62.98% |
| *rps14* | zLOST | Correct_HDR | 2657763 | 0.178610596 | 17.86% |
| *rps14* | zLOST | Incorrect_HDR | 258590 | 0.017378116 | 1.74% |
| *rps14* | zLOST | WT | 257224 | 0.017286316 | 1.73% |
| *rps14* | zLOST | Others | 11706632 | 0.78672497 | 78.67% |
