# Supplementary material for: CRISPR/Cas9-mediated precise genome modification by a long ssDNA template in zebrafish
Source: BMC Genomics. 2020 Jan 21;21:67. doi: 10.1186/s12864-020-6493-4 (PMC6974980; doi:10.1186/s12864-020-6493-4)
Supplement: Supplementary file 5 — Additional file 5: Table S3. Target sites in this study. Red colors means PAM sequences. [file 12864_2020_6493_MOESM5_ESM.docx]

Table S3. Target sites in this study. Red colors means PAM sequences.

| Gene name | Target sites |
| --- | --- |
| *tyr* | GGACTGGAGGACTTCTGGGGAGG |
| *tyr ^25del/25del^* | gTGGACTGGAGGACTTCTGCTGG |
| *rps14* | GAAGAGCAGGTCATCAGCCTAGG |
| *th* | GGGTGATCCTGATCCAGATCCGG |
| *nop56* | GGGGTGTCCGATGCTAAGCTTGG |
| *twist2* | gCCTGGCGAACGTACGCGAGCGG |
| *rpl18* | gCTTCAACAAGGTTATTCTGAGG |
